# Supplementary figures and images for: Blocking Muscarinic Receptor 3 Attenuates Tumor Growth and Decreases Immunosuppressive and Cholinergic Markers in an Orthotopic Mouse Model of Colorectal Cancer
Source: Int J Mol Sci. 2022 Dec 29;24(1):596. doi: 10.3390/ijms24010596 (PMC9820315; doi:10.3390/ijms24010596)

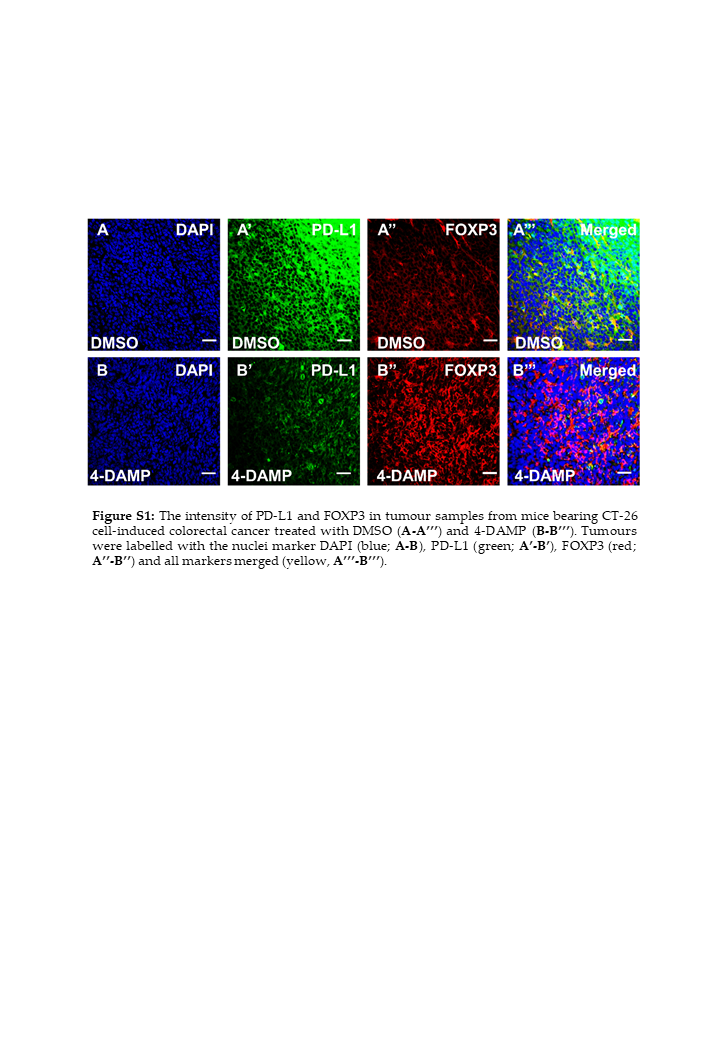

Supplement: Supplementary file 1 [file ijms-24-00596-s001.zip › Supplementary Figure S1 with caption.TIF]

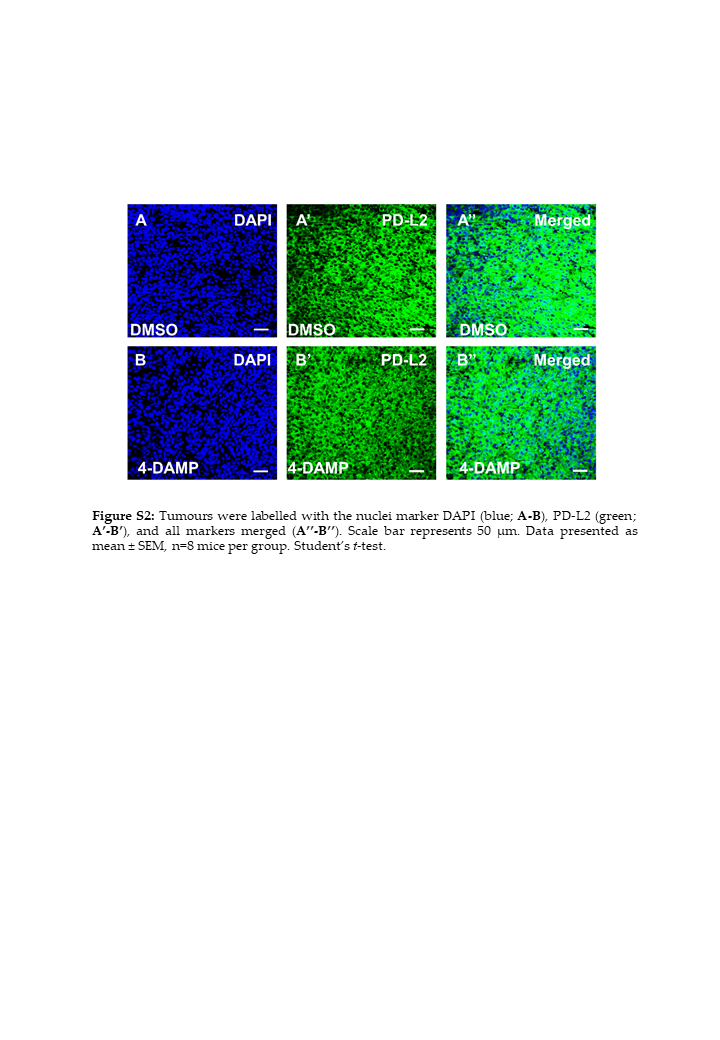

Supplement: Supplementary file 1 [file ijms-24-00596-s001.zip › Supplementary Figure S2 with caption.TIF]

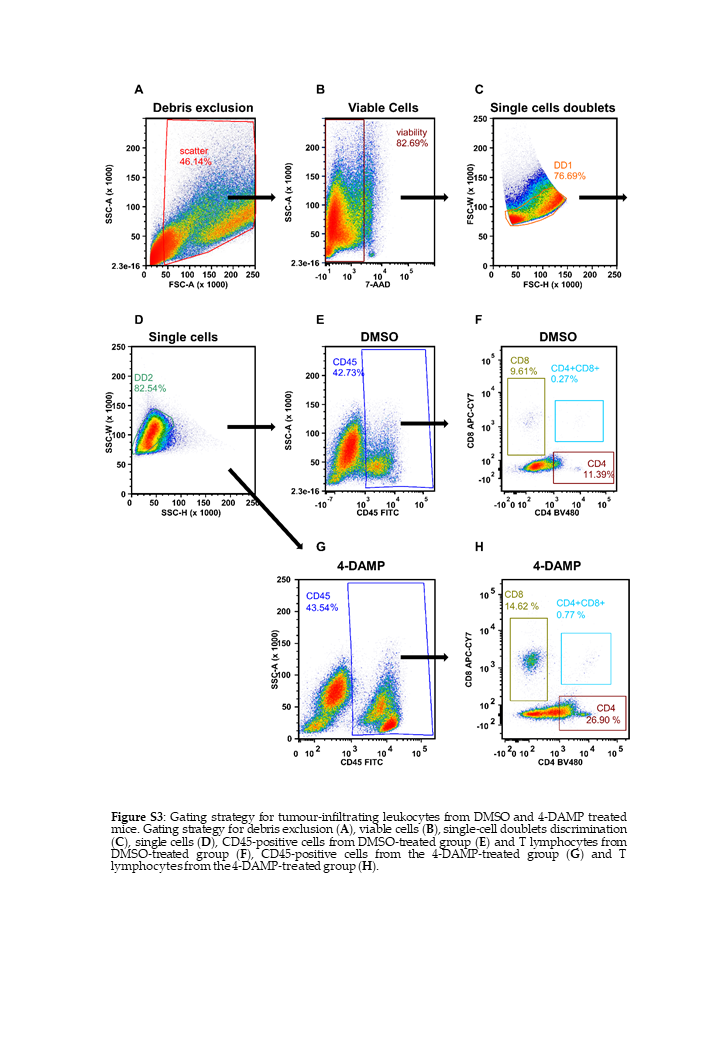

Supplement: Supplementary file 1 [file ijms-24-00596-s001.zip › Supplementary Figure S3 with caption.tif]
